# The Curcumin Analogue PAC Induces Selective Apoptosis-Related Transcriptomic Reprogramming in Oral Squamous Carcinoma Cells

**DOI:** 10.3390/life16071041

**Published:** 2026-06-23

**Authors:** Sara Benchekroun, Meriem Hammache, Fatiha Chandad, Mikhlid H. Almutairi, Adam Daich, Mohammed Badwelan, Mahmoud Rouabhia, Abdelhabib Semlali

**Affiliations:** 1 Groupe de Recherche en Écologie Buccale, Faculté de Médecine Dentaire, Université Laval, Quebec, QC G1V 0A6, Canada; sara.benchekroun.1@ulaval.ca (S.B.); meriem.hammache.1@ulaval.ca (M.H.); fatiha.chandad@greb.ulaval.ca (F.C.); mahmoud.rouabhia@fmd.ulaval.ca (M.R.); 2 Department of Zoology, College of Science, King Saud University, P.O. Box 2455, Riyadh 11451, Saudi Arabia; malmutari@ksu.edu.sa; 3 URCOM UR 3221, Université Le Havre Normandie, Normandie Université, 76600 Le Havre, France; adam.daich@univ-lehavre.fr; 4 2INC3M, FR, CNRS 3038, 25 Rue Philippe Lebon, BP 1123, 76063 Le Havre, France; 5 Faculty of Dental Medicine and Oral Health Sciences, McGill University, Montreal, QC H3A 0G4, Canada; mohammed.badwelan@mail.mcgill.ca

**Keywords:** PAC, curcumin analog, oral squamous carcinoma, apoptosis, transcriptomic profiling, caspases, selective anticancer activity

## Abstract

This study aimed to investigate the selective anticancer activity of the curcumin analog PAC (3,5-Bis-4-hydroxy-3-methoxybenzylidene)-N-methyl-4-piperidone). Normal gingival epithelial cells (GECs), cancerous gingival cells (Ca9-22) and tongue squamous carcinoma cells (CAL27) were exposed to increasing concentrations of PAC (0–10 µM) for 24 h. Cell viability and cytotoxicity were evaluated using MTT and LDH assays, while apoptosis and caspase activation were analyzed by Annexin V/PI staining and flow cytometry. Gene-expression profiling was performed using RT^2^ Profiler PCR arrays. PAC significantly inhibited Ca9-22 and CAL27 cell proliferation in a concentration-dependent manner, with an IC_50_ value of 5 µM, while exerting no noticeable cytotoxic effects on normal GEC. PAC treatment induced significant early and late apoptosis associated with increased caspase activity in both oral cancer cell lines. Transcriptomic analyses revealed extensive modulation of apoptosis-related genes. In Ca9-22 cells, PAC predominantly suppressed anti-apoptotic and survival-associated genes, including BCL2, BIRC3, BIRC5, XIAP, CFLAR, and NFKB1. In contrast, CAL27 cells exhibited a more pronounced pro-apoptotic transcriptional profile characterized by upregulation of TP53, APAF1, CASP1, BID, and TNF. Gene interaction network analyses further demonstrated that PAC targets highly interconnected apoptotic signaling pathways. Collectively, these findings demonstrate that PAC exerts potent selective anticancer activity against OSCC cells through modulation of intrinsic and extrinsic apoptotic pathways. These results further support the therapeutic potential of PAC as a promising multitarget candidate for oral cancer treatment.

## 1. Introduction

Oral cancer (OC) has witnessed a steady rise on a global scale over the past decade, frequently manifesting in advanced clinical stages [1]. It ranks among the ten most prevalent cancers and represents the sixth most common malignancy globally [2,3]. Among all types, oral squamous cell carcinoma (OSCC) remains the predominant histological form, characterized by a high risk of metastasis and late-stage detection [3,4].

Primary preventable risk factors include tobacco use and alcohol consumption, both of which act synergistically to increase mutagenic pressure in oral epithelial tissues [4]. Reports indicate a potential 40% surge in the burden of OSCC by 2040, with no decline anticipated [5]. OC is a multifactorial disease arising from genetic and epigenetic alterations that activate oncogenic pathways while concurrently inactivating tumor suppressor mechanisms, enabling uncontrolled proliferation and immune evasion.

Numerous molecular alterations have been documented in oral carcinogenesis, including mutations inepidermal growth factor (EGFR) [6], Cyclin D1 [7], Rb (retinoblastoma) [8], CDKN2a (cyclin-dependent kinase inhibitor 2a) [9], RAS/RAF/MAPK [10], TP53 [11], among others. These genetic disturbances sustain aberrant proliferation and invasion [5]. The conventional management generally involves surgical resection, followed by adjuvant radiotherapy or chemotherapy. Cisplatin is commonly used as a frontline chemotherapeutic agent, although its non-selective toxicity limits its long-term clinical benefit [5]. However, its indiscriminate nature contributes to various adverse reactions, including hearing impairment, compromised immune function, and renal complications [12].

Although chemotherapy remains essential for inoperable oral cancers, chemoresistance frequently develops, reducing its effectiveness and resulting in treatment failure [5,13]. Epigenetic reprogramming and cellular adaptation are critical in this process. To overcome such limitations, combined strategies, often integrating natural bioactive compounds, are increasingly explored [14,15]. One promising compound is PAC (3,5-Bis-(4-hydroxy-3-methoxybenzylidene)-N-methyl-4-piperidone), a synthetic analog of curcumin. Previous investigations from our group demonstrated that PAC reduces proliferation, enhances apoptosis, and promotes DNA repair in oral cancer cells [5,13]. Moreover, co-administration of PAC with cisplatin resulted in synergistic inhibition of tumor cell growth and an increase in apoptotic signaling**.** This combination reduced the IC_50_ of cisplatin tenfold, suggesting a strong potential for therapeutic synergy.

While previous studies demonstrated that PAC exerts cytotoxic, apoptotic, and autophagic effects in oral cancer cells, the molecular mechanisms underlying its selective anticancer activity remain incompletely understood. The present study therefore investigates the apoptosis-related transcriptomic alterations induced by PAC in oral squamous carcinoma cells. Using RT^2^ Profiler PCR arrays and gene interaction network analyses, we demonstrate that PAC selectively induces apoptosis and extensively modulates intrinsic and extrinsic apoptotic signaling pathways in Ca9-22 and CAL27 cells. Collectively, these findings provide novel mechanistic insight into the selective anticancer activity of PAC and support its potential as a promising multitarget therapeutic candidate against oral squamous cell carcinoma.

## 2. Materials and Methods

### 2.1. Cells Culture

Primary human gingival epithelial cells (GECs) were obtained from Dr Mahmoud Rouabhia’s laboratory. These cells were isolated from healthy human gingival tissues following enzymatic treatment with thermolysin at 500 mg/mL to separate the epithelium from the lamina propria as previously reported in our established protocols [16,17]. GECs were grown in DMEM/F12 medium (Thermo Fisher Scientific), supplemented with 10% of FBS. All cell lines were maintained in a 5% CO_2_ atmosphere at 37 °C in a humidified 5% CO_2_ atmosphere.

Ca9-22 cells, a cell line of human gingival epithelial carcinoma cells, were obtained from the RIKEN BioResource Research Center (Tsukuba, Japan). These cells were cultured in RPMI-1640 medium (Gibco, Thermo Fisher Scientific, Waltham, MA, USA), supplemented with 5% fetal bovine serum (FBS) (Thermo Fisher Scientific) and 1% penicillin-serum, along with 1% Fungizone from Sigma (Oakville, ON, Canada).

CAL 27, a human epithelial cell line derived from a tongue tumor was obtained from ATCC. These cells were cultured in Dulbecco’s Modified Eagle’s Medium (DMEM) modified with 4 mM L-glutamine and 4500 mg/L glucose and supplemented with 10% FBS.

### 2.2. PAC Treatment and Experimental Design

PAC (3,5-Bis(4-hydroxy-3-methoxybenzylidene)-N-methylpiperidin-4-one) was kindly provided by Dr. Ibrahim Al-Jammaz (King Faisal Specialist Hospital and Research Center, Riyadh, Saudi Arabia). PAC was dissolved in DMSO at 10 mM and stored at −20 °C until use. For all experiments, cells were treated with increasing concentrations of PAC (0–10 µM), while 5 µM was used for transcriptomic analyses, corresponding approximately to the IC_50_ concentration previously determined in oral cancer cells [17].

### 2.3. Cell Viability by MTT Assay

Cell viability was evaluated using the MTT assay. Approximately 1.5 × 10^5^ cells per well were seeded into 12-well plates and allowed to adhere overnight. The cells were then exposed to 5 μM PAC for 24 h. Post-treatment cell proliferation was quantified using the MTT assay, following established protocols [18]. Following treatment, 100 µL of MTT solution (5 mg/mL, Sigma) diluted 1:10 (*v*/*v*) in medium was added to each well and incubated at 37 °C for 3 h. The formazan crystals formed were dissolved in isopropanol containing 0.04 M HCl, and absorbance was measured at 550 µM using a Bio-Rad xMark microplate spectrophotometer (Bio-Rad Laboratories, Mississauga, ON, Canada). Cell viability was expressed as a percentage relative to controls, calculated using the formula: % cell viability = [(OD550 of treated cells) − (OD550 of blank)]/[(OD550 of control cells) − (OD550 of blank)] × 100. Each experiment was conducted in triplicate and repeated six times to ensure reproducibility.

### 2.4. Cell Toxicity by LDH Assay

Cytotoxicity was quantified by measuring lactate dehydrogenase (LDH) release into the culture medium. Ca9-22, CAL27 and GECs (1.5 × 10^5^ cells/well) were treated with 5 µM PAC for 24 h, while untreated cells served as controls. LDH activity in the supernatant was determined using a commercial LDH detection kit (Sigma Aldrich, Oakville, ON, Canada) according to the manufacturer’s instructions [19]. Triton X-100 was used to induce complete cell lysis and served as a positive control (100% cytotoxicity). All experiments were performed in triplicate and repeated on six separate occasions.

### 2.5. Annexin V/Propidium Iodide Assay

Apoptosis was analyzed using an APC Annexin V/propidium iodide (PI) staining assay from Biolegend (San Diego, CA, USA) as previously described by Semlali et al. [14,18,20,21]. Ca9-22 and CAL27 cells were cultured overnight to allow adherence. Following adherence, the cells were treated with 5 μM PAC for 24 h at 37 °C, harvested by trypsinisation, and washed twice with PBS. Cells were resuspended in 100 μL of Annexin V-binding buffer and stained with 5 μL of APC Annexin V and 5 μL of PI, incubating for 30 min at room temperature in the dark. Flow cytometric analysis was performed using LSRII or CantoII cytometers from BD Biosciences (Mississauga, ON, Canada) to determine the proportion of cells in various apoptotic stages. This procedure was independently replicated four times.

### 2.6. Caspase Activity

Caspase activation was measured using a FITC-VAD-FMK Caspase Detection Kit (Millipore, Burlington, MA, USA). Following PAC treatment (5 µM, 24 h), cells were incubated with FITC-VAD-FMK reagent for 1 h at 37 °C, washed, and analyzed by flow cytometry (FL1 channel). Data were expressed as the percentage of caspase-positive cells. Experiments were performed independently in triplicate.

### 2.7. RNA Extraction from Oral Cancer Cells

Cells were lysed in Trizol reagent (Sigma Aldrich) and stored at −80 °C. RNA extraction followed the manufacturer’s standard protocol. The aqueous phase was collected after chloroform separation, and RNA was precipitated with isopropanol, washed in 75% ethanol, and dissolved in RNase-free water. RNA purity and concentration were assessed by NanoDrop spectrophotometry (Thermo Fisher, Waltham, MA, USA).

### 2.8. cDNA Synthesis and RT^2^ Profiler PCR Arrays

Complementary DNA (cDNA) was synthesized from 2 µg of total RNA using the RT^2^ First Strand Kit (Qiagen, Toronto, ON, Canada). Gene expression related to apoptosis was analyzed using Qiagen RT^2^ Profiler PCR Arrays (Human Apoptosis PAHS-0127Z) (Toronto, ON, Canada). Each PCR reaction contained 1350 µL of RT^2^ SYBR Green Master Mix, 102 µL of cDNA, and 1248 µL of RNase-free water. The reactions were run on a CFX-96 Real-Time PCR System (Bio-Rad), and relative gene expression was calculated using the 2^−ΔΔCT^ method. Only genes showing at least a two-fold change compared with the control were considered differentially expressed.

### 2.9. Gene–Gene Interaction Analysis

Functional gene-interaction networks were generated using the Gene MANIA online platform. The analysis included co-expression, physical interaction, and pathway association data. Genes sharing similar functions were displayed in the outer circle, whereas hub genes were placed in the center.

### 2.10. Statistical Analysis

The experiments were conducted independently at least three times. Results are presented as mean ± standard deviation (SD). Comparisons between control and PAC-treated groups were analyzed by one-way ANOVA followed by Tukey’s post hoc test, with *p* < 0.05 considered statistically significant. Additional analyses were performed using SAS v8.2 (SAS Institute, Cary, NC, USA) and FCS Express de Novo software 7 for flow cytometry data. MTT and LDH data were analyzed using Student’s *t*-test in Microsoft Excel. Gene expression analysis via qPCR array was performed using the 2^−ΔΔCt^ (Livak) relative expression method to compare gene expression levels and fold changes between untreated cells and those treated with PAC. Gene expression normalization was achieved through an automated process using a comprehensive set of reference genes. Control samples consisted of untreated cells, while the experimental group included cells treated with 5 μM of PAC (IC50). CT values were compiled into a table, which was subsequently uploaded to the web portal for data analysis, accessible at http://www.qiagen.com/geneglobe (accessed on 26 May 2026).

## 3. Results

### 3.1. PAC Emerges as a Promising Alternative Drug Candidate for Oral Cancer Therapy by Its Capacity Selectively Target Oral Cancer Cells

**Figure 1:**
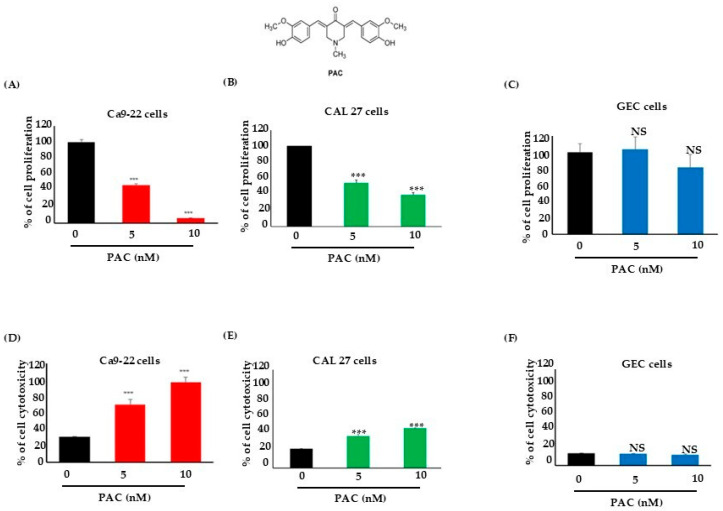
PAC inhibits specifically oral cancer cell growth and induces cell cytotoxicity but not in normal cells. (**A**) MTT assay in Ca9-22 cells after PAC treatment for 24 h (n = 6). (**B**) MTT assay in CAL27 cells after PAC treatment for 24 h (n = 5). (**C**) Effect of PAC treatment on GEC proliferation by MTT assay (n = 4). (**D**) Effect of PAC treatment for 24 h on Ca9-22 cell cytotoxicity performed by LDH (n = 6). (**E**) LDH assay for CAL 27 (n = 5) and (**F**) LDH assay for GECs treated by PAC treatment for 24 h (n = 4). All data was presented as the mean of percentage ± SEM. *** Correspond to *p* < 0.0005.

To assess the impact of PAC on cell viability and toxicity, MTT and LDH assays were used to evaluate the effect of PAC on Ca9-22 cells, CAL27 (representing tumor cells) and GECs (representing normal cells) after 24 h of treatment. PAC treatment resulted in a clear concentration-dependent reduction in Ca9-22 and CAL27 cell viability, whereas normal GECs remained largely unaffected (Figure 1A–C).

At concentrations of 5 and 10 µM, PAC significantly inhibited the proliferation of Ca9-22 cells and CAL27 (*p* < 0.001), while no significant change was observed in GEC cell viability. These findings confirm that PAC exerts selective cytotoxicity against oral cancer cells while preserving normal epithelial cell integrity.

The cytotoxicity of PAC was further quantified by LDH release into the culture medium. Untreated cells exhibited minimal LDH release, confirming membrane integrity, whereas PAC-treated Ca9-22 cells and CAL27 showed a significant increase in LDH activity (*p* < 0.01) (Figure 1D,E). In contrast, LDH activity in GECs exposed to PAC treatment remained comparable to that of controls, indicating the absence of cytolytic damage (Figure 1F).

### 3.2. PAC Exerts Its Anti-Oral Cancer Activity by Triggering Cell Apoptosis Through Caspases Activity

**Figure 2:**
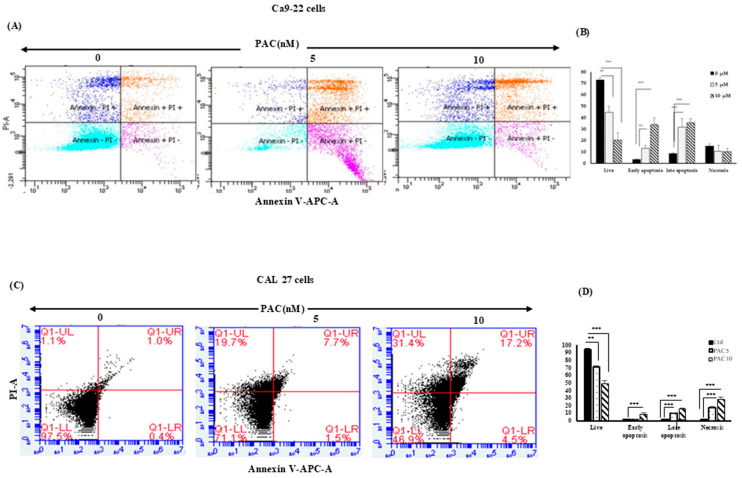
PAC treatment promotes apoptosis in Ca9-22 and CAL27 cells. (**A**) Flow cytometry by using APC Annexin V/PI array was performed in Ca9-22 cells (n = 4 experiments). Data was presented as a means of percentage ± SD of cells in different stages of apoptosis (early, late apoptosis and necrosis phase). (**B**) Representative data from four independent experiments in Ca9-22 cells presented by mean ± SD values. (**C**) Flow cytometry data CAL-27 cells (n = 3 experiments). (**D**) Representative data from three independent experiments in CAL 27 cells presented by mean ± SD values. ** *p* < 0.001 and *** *p* < 0.0001.

**Figure 3:**
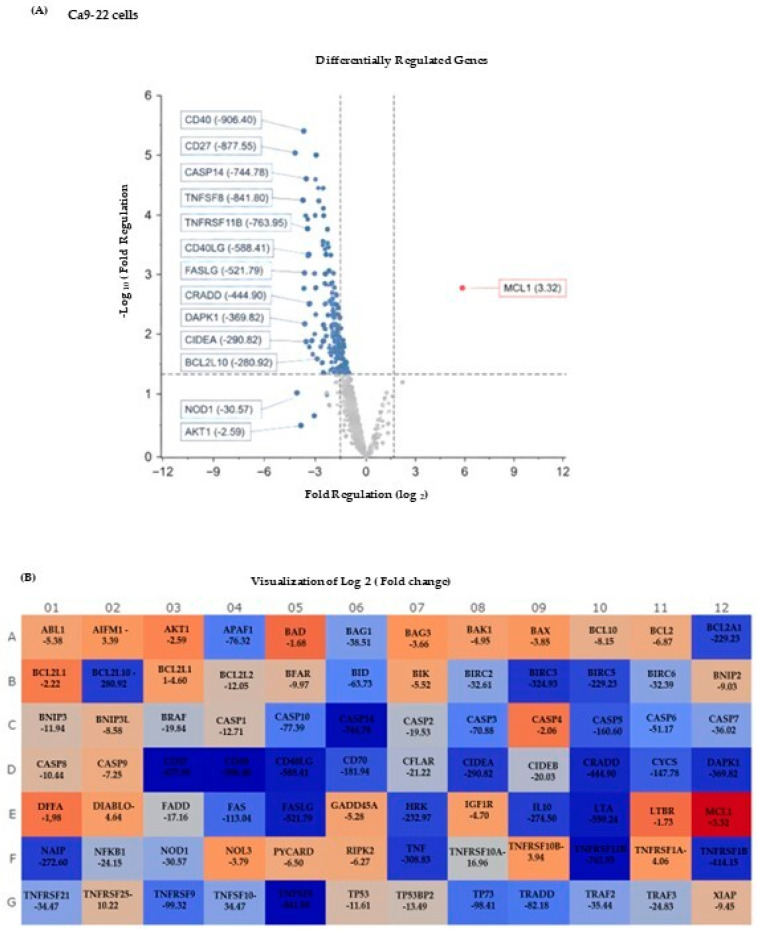

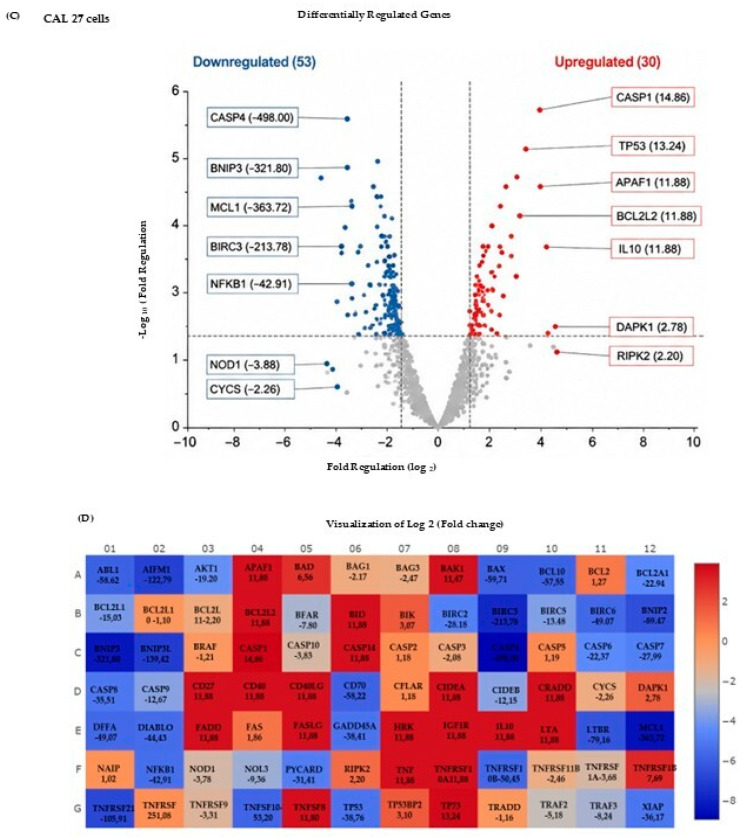

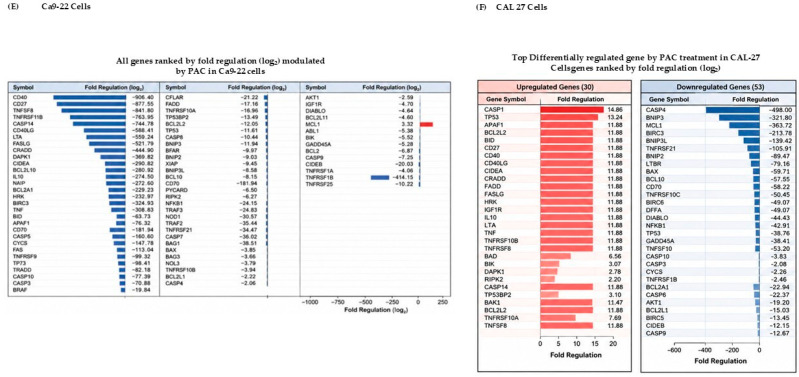
(**A**–**D**) PAC-mediated modulation of apoptosis-related genes in Ca9-22 and CAL27 oral cancer cells identified using RT^2^ Profiler PCR Array. Volcano plots illustrate differentially expressed apoptosis-associated genes following treatment with 5 µM PAC. The central diagonal line represents unchanged gene expression, whereas the outer diagonal lines indicate the two-fold regulation threshold. Red symbols represent upregulated genes, and blue symbols represent downregulated genes. Heat maps summarize the expression profiles of apoptosis-related genes in both cell lines following PAC treatment (n = 3). (**E**,**F**) Ranked representation of the most significantly upregulated and downregulated apoptosis-related genes in Ca9-22 and CAL27 cells following PAC exposure.

To validate the anti-cancer efficacy of PAC human squamous cell carcinoma cell line (Ca9-22 and CAL 27 cells), we examined its ability to induce apoptosis, a well-established mechanism for eliminating cancer cells. As depicted in Figure 2A–D, flow cytometry analysis using the APV Annexin V/PI assay revealed that PAC induces apoptosis in oral cancer cells after 24 h treatment with concentration as low as 5 µM of PAC. In addition, the percentage of viable cells decreases dose-dependent manner with PAC treatment declining from 72.8% ± 2.2% to 50.25% ± 5.05% with 5 µM of PAC and to 20.4% ± 6.4% with 10 µM of PAC on Ca9-22 cells (Figure 2A,B), while the percentage of viable cells decreases from 94.4% ± 1.59% from CAL 27 untreated cells to 71.1% ± 1.6% and to 46.9% ± 4.26% when CAL 27 cells were treated respectively by 5 and 10 µM of PAC (Figure 2C,D). Additionally, the percentage of early apoptotic cells increased significantly from 3.5% ± 0.4% in control cells to 12.55% ± 2.75% and 34.00% ± 5.8% in Ca9-22 cells treated cells with 5 and 10 µM of PAC respectively (Figure 2A,B). The % of early apoptosis increases in CAL 27 cells from 1.7% ± 0.67% to 8.23% ± 1.87% with PAC- treatment at 10 µM (Figure 2C,D). Furthermore, the percentage of late apoptotic cells rose in Ca9-22 cells from 8.45% ± 0.85% in control cells to 26.75% ± 7.45% and 35.5% ± 3.7% post-PAC treatment with 5 and 10 µM respectively (Figure 2A,B). This percentage of late apoptosis increases in CAL 27 cells from 1.95% ± 0.45% to 9.53%± 1.23% and 15.33% ± 1.39% with PAC treatment at respectively 5 and 10 µM (Figure 3C,D). These findings strongly support the notion that PAC holds promise as a therapeutic agent for targeting oral cancer cells through the induction of apoptosis.

Caspase activation was confirmed by FITC-VAD-FMK staining. PAC-treated Ca9-22 and CAL 27 cells showed significantly higher fluorescence intensity compared with untreated controls (*p* < 0.001), indicating increased caspase activity. The enhanced caspase response supports the hypothesis that PAC induces apoptosis through caspase-dependent apoptotic pathways.

### 3.3. PAC Induces Apoptosis-Related Transcriptional Reprogramming in Both Ca9-22 and CAL27 Oral Cancer Cells

To further investigate the molecular mechanisms underlying the anticancer activity of PAC, the expression profile of 84 apoptosis-related genes was analyzed using the RT^2^ Profiler PCR Array in both Ca9-22 and CAL27 oral squamous cell carcinoma (OSCC) cell lines following treatment with 5 µM PAC. Overall, PAC induced profound transcriptional reprogramming of apoptosis-associated pathways in both cellular models, although the response patterns differed between the two cell lines.

In Ca9-22 cells, PAC modulated 81 of the 84 apoptosis-related genes analyzed. Among these, 80 genes were significantly downregulated, with fold changes ranging from −2-fold to more than −900-fold (Figure 3A,B). Only one anti-apoptotic gene, myeloid cell leukemia 1 (MCL1), was upregulated following PAC treatment (+3.32-fold). The most strongly downregulated genes included CD40 (−906.40-fold), CD27 (−877.55-fold), TNFRSF1B (−841.80-fold), TNFRSF11B (−763.95-fold), CASP14 (−744.78-fold), CD40LG (−588.41-fold), and FASLG (−521.79-fold).

A large proportion of the modulated genes were associated with anti-apoptotic signaling pathways. These included AKT1 (−2.59), BAG1 (−38.58), BAG3 (−3.66), BCL2 (−6.87), BCL2A1 (−229.23), BCL2L10 (−280.92), BCL2L2 (−4.60), BIRC3 (−324.93), BIRC5 (−229.23), BIRC6 (−32.39), BFAR (−9.97), HRK (−232.97), IGF1R (−4.70), IL10 (−274.50), NAIP (−272.60), NFKB1 (−24.15), RIPK2 (−6.27), TNF (−308.83), and XIAP (−9.45). Additional apoptosis suppressors, including BCL10 (−8.15), BIRC2 (−32.39), CIDEA (−290.82), TP53 (−11.61), and TP73 (−98.14), were also significantly reduced.

PAC treatment also markedly affected the caspase signaling network in Ca9-22 cells. Eleven caspase genes were significantly downregulated, including CASP1 (−12.71-fold), CASP2 (−19.53-fold), CASP3 (−70.88-fold), CASP5 (−160.60-fold), CASP6 (−51.17-fold), CASP7 (−36.02-fold), CASP8 (−10.44-fold), CASP9 (−7.25-fold), CASP10 (−77.39-fold), and CASP14 (−744.78-fold). Similarly, genes involved in death receptor-mediated apoptosis signaling, including FADD (−17.16-fold), TRADD (−82.18-fold), TNFRSF10A (−16.96-fold), TNFRSF10B (−3.94-fold), TNFRSF11B (−763.95-fold), TNFRSF1A (−4.05-fold), TNFRSF1B (−414.15-fold), TNFRSF21 (−34.47-fold), and TNFRSF25 (−10.22-fold), were strongly inhibited. Collectively, these data suggest that PAC profoundly suppresses survival, inflammatory, and death receptor-associated signaling pathways in Ca9-22 cells, which may contribute to apoptotic cell death and suppression of survival signaling pathways.

In contrast, CAL27 cells exhibited a distinct transcriptional response to PAC treatment. Among the 84 apoptosis-related genes analyzed, PAC significantly modulated 83 genes, including 53 downregulated and 30 upregulated genes (Figure 3C,D). Notably, several classical pro-apoptotic genes were markedly induced, including CASP1 (+14.86-fold), TP53 (+13.24-fold), APAF1 (+11.88-fold), BCL2L2 (+11.88-fold), IL10 (+11.88-fold), DAPK1 (+2.78-fold), and RIPK2 (+2.20-fold). Other upregulated genes included BID (+11.88-fold), CD40 (+11.88-fold), CD40LG (+11.88-fold), CRADD (+11.88-fold), FADD (+11.88-fold), FASLG (+11.88-fold), IGF1R (+11.88-fold), TNF (+11.88-fold), TNFRSF10B (+11.88-fold), and TNFSF8 (+11.88-fold).

Conversely, several anti-apoptotic and survival-related genes were strongly downregulated in CAL27 cells, including CASP4 (−498.00-fold), BNIP3 (−321.80-fold), MCL1 (−363.72-fold), BIRC3 (−213.78-fold), BNIP3L (−139.42-fold), TNFRSF10C (−50.45-fold), BIRC6 (−49.07-fold), NFKB1 (−42.91-fold), GADD45A (−38.41-fold), BCL2A1 (−22.37-fold), BIRC5 (−13.45-fold), and CASP9 (−12.67-fold). These findings indicate that, unlike Ca9-22 cells, CAL27 cells respond to PAC by simultaneously activating multiple pro-apoptotic mediators while suppressing critical survival pathways.

Overall, the RT^2^-PCR array analyses demonstrate that PAC exerts a potent modulatory effect on apoptosis-associated signaling in OSCC cells. Importantly, the transcriptional responses differed substantially between Ca9-22 and CAL27 cells, highlighting a cell line-dependent mechanism of action. While PAC predominantly induced widespread transcriptional repression in Ca9-22 cells, it triggered a mixed pro-apoptotic transcriptional activation profile in CAL27 cells, particularly through the induction of TP53-, APAF1-, and caspase-related pathways.

### 3.4. Gene–Gene Interaction Network Analysis

**Figure 4:**
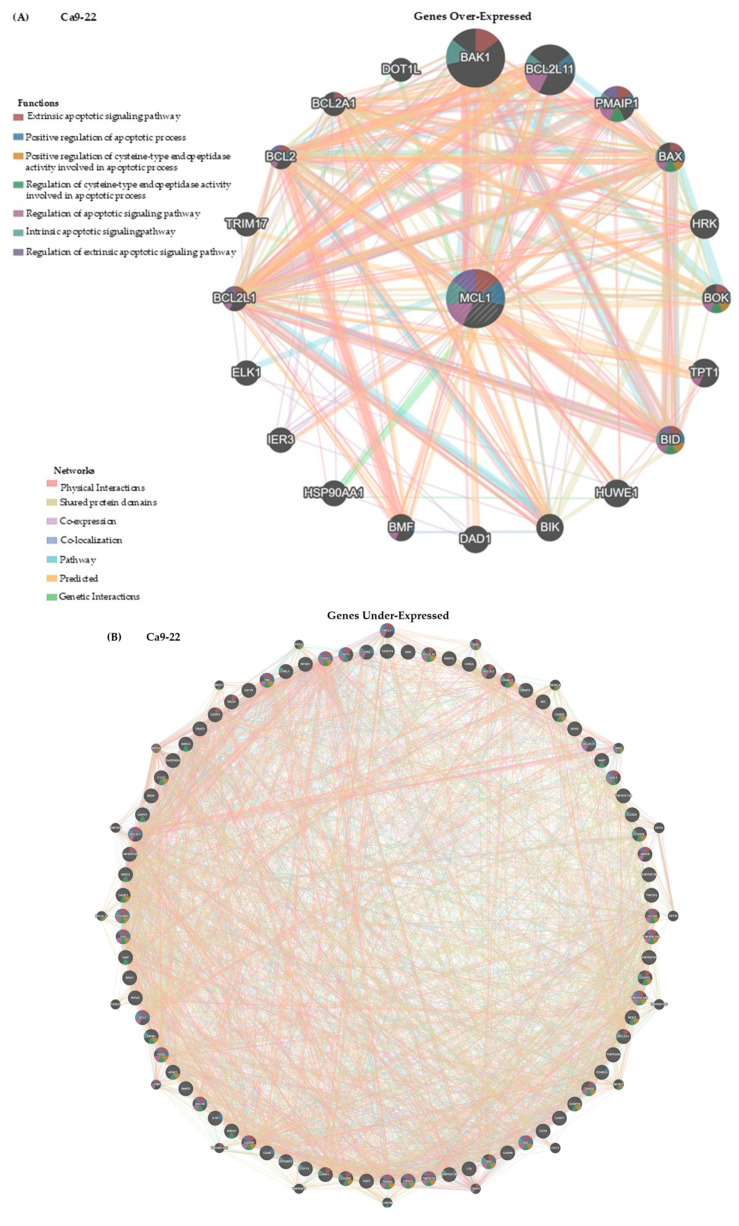

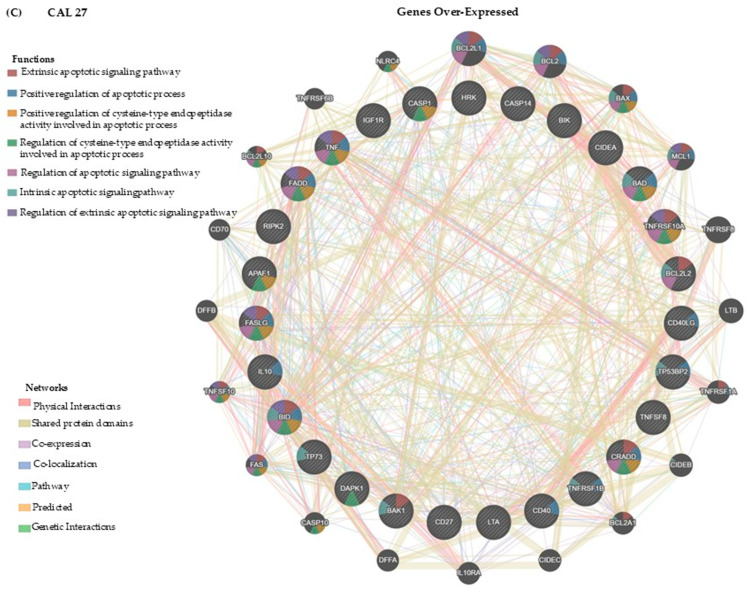

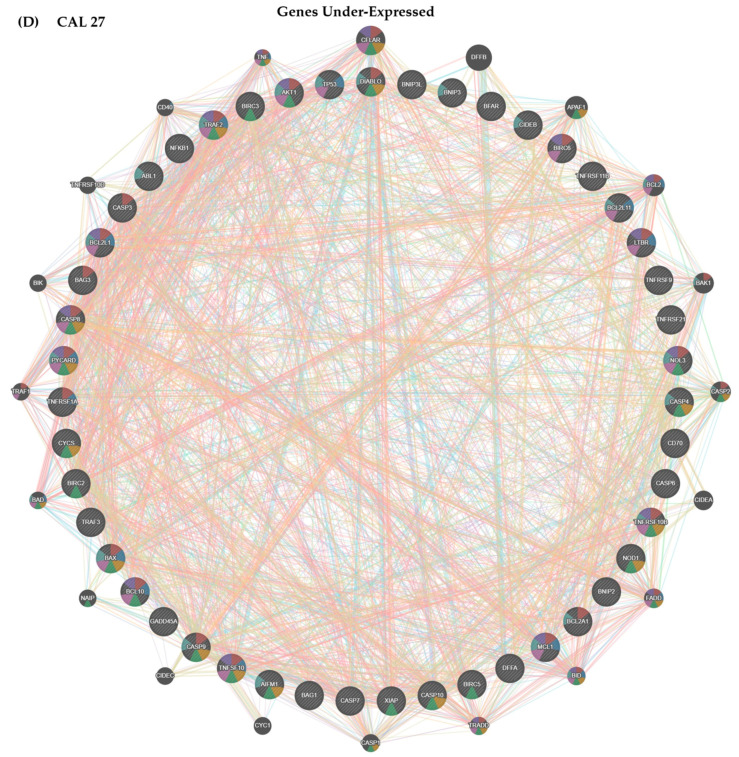
Gene–gene interaction networks of apoptosis-related genes modulated by PAC in (**A**,**B**) Ca9-22 and (**C**,**D**) CAL27 oral cancer cells. Networks were generated using differentially expressed genes identified by RT^2^ Profiler PCR Array analysis. Nodes represent genes/proteins, whereas edges indicate predicted or experimentally validated interactions, including physical interactions, co-expression, co-localization, pathway associations, shared protein domains, and genetic interactions. Functional enrichment analyses revealed significant associations with intrinsic and extrinsic apoptotic signaling pathways, regulation of apoptotic processes, and caspase-associated signaling cascades. Distinct network architectures were observed between Ca9-22 and CAL27 cells following PAC treatment.

To further elucidate the molecular mechanisms underlying PAC-mediated apoptosis in oral squamous cell carcinoma (OSCC), gene–gene interaction network analyses were performed using the significantly modulated apoptosis-related genes identified in Ca9-22 and CAL27 cells following PAC treatment. The resulting interaction networks revealed complex regulatory relationships between apoptosis-associated genes, cell cycle regulators, inflammatory mediators, and DNA damage response pathways (Figure 4).

The generated networks consisted of interconnected gene nodes and interaction edges representing multiple types of biological associations, including physical interactions, co-expression, co-localization, shared protein domains, predicted interactions, pathway associations, and genetic interactions. Highly interconnected hub genes were identified in both cell lines, suggesting that PAC induces extensive transcriptional reprogramming affecting several key oncogenic and apoptotic signaling pathways simultaneously.

In Ca9-22 cells, PAC treatment predominantly induced a global suppression of apoptosis-related signaling networks (Figure 4A,B). Most of the modulated genes were significantly downregulated, including major anti-apoptotic regulators such as BCL2, BCL2A1, BCL2L10, BIRC3, BIRC5, XIAP, NFKB1, and AKT1. The network analysis further demonstrated strong interactions between genes involved in TNF/TNFR-mediated signaling, inflammatory responses, and caspase regulation. Highly connected nodes included TNF, TNFRSF family members, CASP3, CASP8, CASP10, FADD, TRADD, and CFLAR, indicating that PAC strongly disrupts extrinsic apoptotic signaling pathways in Ca9-22 cells. In addition, several genes involved in cell survival and resistance to apoptosis, including IGF1R, HRK, BAG1, and RIPK2, were integrated within the same interaction clusters, suggesting coordinated inhibition of pro-survival pathways.

Interestingly, the network topology observed in CAL27 cells differed markedly from that of Ca9-22 cells (Figure 4C,D). In CAL27 cells, PAC induced a mixed transcriptional response characterized by simultaneous activation of several pro-apoptotic genes and suppression of survival-related pathways. Central hub genes included TP53, APAF1, CASP1, BID, FADD, TNF, and DAPK1, which formed highly interconnected apoptotic signaling modules associated with intrinsic and extrinsic apoptosis pathways. Functional enrichment analysis demonstrated that these genes were strongly associated with positive regulation of apoptotic processes, regulation of cysteine-type endopeptidase activity, and apoptotic signaling cascades. Conversely, several anti-apoptotic and tumor-promoting genes, including MCL1, BIRC3, BIRC5, BIRC6, NFKB1, and BCL2A1, were markedly downregulated and formed interconnected survival-associated subnetworks.

The interaction maps also highlighted the central role of inflammatory and death receptor signaling in PAC-mediated responses. Genes associated with TNF superfamily signaling, including TNF, TNFRSF10B, FASLG, CD40, and CD40LG, displayed extensive connectivity with caspase-associated pathways, suggesting that PAC may promote apoptosis through coordinated activation of death receptor-mediated mechanisms, particularly in CAL27 cells.

Moreover, pathway enrichment analysis revealed that the modulated genes in both cell lines were primarily associated with extrinsic apoptotic signaling pathways, intrinsic mitochondrial apoptosis pathways, regulation of apoptotic signaling, and positive regulation of cysteine-type endopeptidase activity involved in apoptosis. The coexistence of multiple interconnected pathways suggests that PAC exerts pleiotropic anticancer effects by simultaneously targeting several molecular regulators controlling apoptosis, inflammation, cell survival, and stress responses.

Overall, these findings demonstrate that PAC profoundly remodels apoptosis-related gene interaction networks in OSCC cells. Importantly, the distinct network architectures observed between Ca9-22 and CAL27 cells indicate a cell line-dependent mechanism of action. While PAC mainly induced widespread transcriptional repression of survival and inflammatory pathways in Ca9-22 cells, it preferentially activated pro-apoptotic signaling hubs in CAL27 cells. These results further support the hypothesis that PAC exerts potent anticancer activity through coordinated modulation of multiple interconnected apoptotic and cell survival pathways. 

## 4. Discussion

Oral squamous cell carcinoma (OSCC) remains one of the most aggressive malignancies of the oral cavity and continues to represent a major public health challenge worldwide. Despite advances in surgery, radiotherapy, and chemotherapy, the prognosis of advanced OSCC remains poor due to tumor recurrence, metastasis, chemoresistance, and the severe toxicity associated with conventional anticancer therapies [1,2,3,4,5]. Cisplatin-based chemotherapy remains one of the principal therapeutic strategies for advanced oral cancers; however, its clinical efficacy is frequently limited by systemic toxicity and the progressive emergence of resistant tumor cells [12,13]. Consequently, the development of selective anticancer agents capable of targeting multiple oncogenic pathways while preserving normal tissues has become increasingly important.

In this context, naturally derived compounds and synthetic analogues have attracted considerable attention because of their multitarget biological activities and reduced toxicity profiles [14,15,16,17,18]. Among these compounds, PAC (3,5-Bis(4-hydroxy-3-methoxybenzylidene)-N-methylpiperidin-4-one), a synthetic curcumin analog, has previously demonstrated promising anticancer properties in several malignancies, including breast, thyroid, colon, and oral cancers [15,16,17,18,19,20,21]. Previous studies from our group demonstrated that PAC suppresses oral cancer cell proliferation and exhibits synergistic effects with cisplatin [15,17]. However, the molecular mechanisms underlying its selective anticancer activity remained incompletely understood. The present study significantly expands previous findings by providing, for the first time, a comprehensive transcriptomic analysis of apoptosis-related gene modulation induced by PAC in two distinct oral squamous carcinoma cell lines, Ca9-22 and CAL27.

One of the major findings of the present study is the selective cytotoxic effect of PAC against oral cancer cells while sparing normal gingival epithelial cells. PAC significantly reduced the viability of both Ca9-22 and CAL27 cells in a concentration-dependent manner, whereas normal gingival epithelial cells remained largely unaffected. These findings are particularly important because selective toxicity toward malignant cells represents a critical requirement for the development of safer anticancer therapies. Similar observations have previously been reported for PAC and other curcumin analogs in different tumor models [16,17,18,19,20,21], supporting the hypothesis that structural modifications of curcumin may substantially improve its therapeutic efficacy and selectivity.

Compared with native curcumin, PAC appears to exhibit improved anticancer potency and greater biological stability, likely due to structural modifications enhancing its bioavailability and intracellular activity. Previous studies demonstrated that PAC induces stronger apoptotic responses than curcumin in several cancer models while maintaining low toxicity toward normal cells. These properties further support the translational potential of PAC as a next-generation curcumin analog for OSCC therapy.

The present work further demonstrates that PAC induces apoptosis in both oral cancer cell lines through caspase-dependent mechanisms. Annexin V/PI flow cytometry analyses revealed significant increases in both early and late apoptotic populations following PAC treatment in Ca9-22 and CAL27 cells. These findings were corroborated by enhanced caspase activity detected using FITC-VAD-FMK staining, confirming activation of apoptotic pathways. Importantly, although apoptosis was induced in both cell lines, the transcriptional responses associated with apoptosis regulation differed markedly between Ca9-22 and CAL27 cells, suggesting a cell line-dependent mechanism of action.

Transcriptomic profiling of apoptosis-related genes revealed that PAC induced profound molecular reprogramming in both oral cancer cell lines. In Ca9-22 cells, PAC predominantly induced widespread downregulation of apoptosis-associated genes. Several anti-apoptotic and survival-related genes, including BCL2, BCL2A1, BCL2L10, BIRC3, BIRC5, BIRC6, XIAP, CFLAR, NFKB1, TNF, and multiple TNF receptor superfamily members, were strongly suppressed following PAC treatment. Many of these genes are known to contribute to tumor progression, resistance to apoptosis, and chemoresistance in various cancers, including OSCC [22,23,24]. The strong inhibition of these survival-associated pathways therefore suggests that PAC exerts its anticancer effects in Ca9-22 cells primarily through suppression of anti-apoptotic signaling networks. Particularly noteworthy was the marked inhibition of members of the inhibitor of apoptosis protein (IAP) family, including BIRC2, BIRC3, BIRC5, and BIRC6. These proteins are recognized as key regulators of cancer-cell survival because they suppress caspase activation and promote resistance to apoptosis [25,26]. Their inhibition by PAC strongly supports the hypothesis that PAC restores apoptotic sensitivity in oral cancer cells. Similarly, the inhibition of CFLAR/c-FLIP, a major suppressor of caspase-8 activation and death receptor signaling, suggests that PAC may facilitate activation of extrinsic apoptotic pathways [27,28].

Interestingly, despite the strong induction of apoptosis observed by flow cytometry and caspase assays, many caspase-related genes were transcriptionally downregulated in Ca9-22 cells following PAC treatment. Interestingly, despite the strong induction of apoptosis observed by flow cytometry and caspase activity assays, several caspase-related genes were transcriptionally downregulated following PAC treatment in Ca9-22 cells. This discrepancy may reflect a secondary transcriptional shutdown occurring during advanced apoptotic progression. Indeed, several studies have demonstrated that apoptosis can involve rapid post-translational activation of caspases followed by generalized repression of transcriptional activity as cells progress toward apoptotic collapse. Therefore, the functional activation of apoptosis observed in our experiments likely reflects activation of pre-existing apoptotic machinery rather than sustained transcriptional induction of caspase genes.

In contrast to Ca9-22 cells, CAL27 cells exhibited a markedly different transcriptomic response following PAC treatment. In these cells, PAC induced simultaneous activation of several classical pro-apoptotic genes, including CASP1, TP53, APAF1, BID, BAK1, DAPK1, TNF, FADD, and TNFRSF10B, while suppressing multiple anti-apoptotic genes such as MCL1, BIRC3, BIRC5, BIRC6, BCL2A1, and NFKB1. The induction of TP53 and APAF1 strongly suggests activation of the intrinsic mitochondrial apoptotic pathway in CAL27 cells. Furthermore, the upregulation of TNF-associated and death receptor-related genes indicates concomitant activation of extrinsic apoptotic signaling pathways.

The differential apoptotic transcriptional signatures observed between Ca9-22 and CAL27 cells highlight the molecular heterogeneity of OSCC and emphasize the complexity of PAC-mediated anticancer activity. While PAC induced a predominantly suppressive apoptotic profile in Ca9-22 cells, it triggered a more classical pro-apoptotic activation program in CAL27 cells. Such differences likely reflect the distinct genetic and epigenetic backgrounds of the two oral cancer cell lines. Importantly, this pleiotropic mechanism of action may represent a considerable therapeutic advantage, as it suggests that PAC can adaptively target multiple apoptotic pathways depending on the molecular characteristics of the tumor.

Gene–gene interaction network analyses further demonstrated that the genes modulated by PAC form highly interconnected apoptotic signaling networks involving inflammatory mediators, death receptor pathways, mitochondrial apoptosis regulators, and caspase-associated cascades. Hub genes such as TP53, TNF, APAF1, CASP3, BIRC5, and NFKB1 displayed extensive interactions with multiple apoptosis-related pathways, highlighting the broad regulatory effects of PAC on oral cancer cell survival. Functional enrichment analyses confirmed strong associations with intrinsic and extrinsic apoptotic signaling pathways, regulation of apoptotic processes, and caspase-mediated cell death. These findings reinforce the hypothesis that PAC exerts multitarget anticancer effects through simultaneous modulation of several interconnected molecular pathways.

Collectively, the present findings demonstrate that PAC possesses potent selective anticancer activity against oral squamous carcinoma cells through coordinated modulation of apoptosis-associated signaling pathways. The ability of PAC to selectively eliminate oral cancer cells while preserving normal epithelial cells, together with its capacity to target multiple anti-apoptotic mechanisms simultaneously, highlights its strong therapeutic potential for OSCC management. Furthermore, the previously reported synergistic effects between PAC and cisplatin [15,17] suggest that PAC may also represent a promising adjuvant agent for combination therapies aimed at overcoming chemoresistance.

Nevertheless, several limitations should be acknowledged. The present work was conducted exclusively in vitro, and additional in vivo studies will be required to validate the therapeutic efficacy, pharmacokinetic properties, and safety profile of PAC in oral cancer models. Although the present study focused on apoptosis-related transcriptomic profiling, future studies should validate key targets identified herein, including TP53, APAF1, BCL2, and BIRC5, at the protein level. It should be noted that several major PAC-regulated proteins, including p53, p21, p27, Bcl-2, Bax, and Cyclin D1, were previously validated in our earlier study [18]. Future investigations should also explore the proteomic and epigenetic mechanisms underlying PAC-mediated apoptosis and further characterize the molecular basis of the differential responses observed between Ca9-22 and CAL27 cells. In addition, gene-specific functional studies using knockdown or overexpression approaches will be required to further validate the contribution of individual apoptosis-related genes and signaling pathways identified in the present transcriptomic analysis” Such studies may help identify predictive biomarkers of PAC sensitivity and optimize its potential clinical application. Although the present study was limited to in vitro investigations, the reproducible anticancer effects observed in both Ca9-22 and CAL27 cells provide a strong rationale for subsequent in vivo validation. Accordingly, the next stage of this research will involve the development of orthotopic and xenograft OSCC models, following the required ethical approvals, to evaluate the therapeutic efficacy, systemic toxicity, pharmacokinetics, and biological effects of PAC within the tumor microenvironment. These studies will be necessary to support the clinical translation of PAC as a potential therapeutic agent for oral squamous cell carcinoma.

## 5. Conclusions

This study demonstrates that the curcumin analog PAC exerts selective anticancer activity against oral squamous cell carcinoma cells by inducing apoptosis-related signaling pathways while sparing normal gingival epithelial cells. PAC promoted caspase-dependent apoptosis and extensively modulated apoptotic gene networks in both Ca9-22 and CAL27 cells, although distinct transcriptomic responses were observed between the two cell lines. These findings highlight PAC as a promising multitarget therapeutic candidate for oral cancer treatment and support further in vivo and translational investigations.

## Data Availability

All data generated in the current study are included in this published article.
